# Limited and inconclusive effects of computer-based neurocognitive training on PTSD, comorbid depression and executive functioning: a systematic review and meta-analysis

**DOI:** 10.3389/fpsyg.2025.1577026

**Published:** 2025-08-13

**Authors:** Patric Muschner, Lena Goldschmidt, Luisa Sophie Gröning, Gerd-Dieter Willmund, Martina Piefke

**Affiliations:** ^1^Neurobiology and Genetics of Behavior, Department of Psychology and Psychotherapy, Witten/Herdecke University, Witten, Germany; ^2^Department of Psychiatry, Psychotherapy, German Armed Forces Hospital, Berlin, Germany

**Keywords:** posttraumatic stress disorder, trauma treatment, depression, affective disorder, executive function training, adjuvant therapy, meta-analysis, systematic review

## Abstract

Patients with posttraumatic stress disorder (PTSD) and comorbid depression often experience neurocognitive deficits. While computer-based neurocognitive training (c-bnt) has shown benefits in primary depression, its efficacy for PTSD remains unclear. This meta-analysis evaluated the impact of c-bnt on PTSD severity, comorbid depression, and neurocognitive functions. A systematic review following PRISMA guidelines was conducted across databases (PsycINFO, PubMed, Scopus, PubPsych) from February to June 2025, focusing on randomized controlled trials (RCTs) involving PTSD and c-bnt. Bias risk was assessed using the revised Cochrane Risk-of-Bias Tool, and effect sizes were calculated with Cohen’s *d*. Heterogeneity was measured using *I*^2^. The review, registered with PROSPERO (CRD42023444417), included eight studies, with five providing meta-analyses data (*n* = 221). Although c-bnt led to a small reduction (*d* = −0.21) on PTSD symptoms, this effect was not statistically significant (*p* = 0.31). Comorbid depression showed inconsistent improvements, with no significant overall effect (*d* = −0.10). A systematic review of neurocognitive functions, covering six studies (*n* = 220), showed mixed results for cognitive flexibility and working memory, but no significant improvements in inhibition. These findings suggest that c-bnt may contribute to reductions in PTSD symptoms and potentially benefit comorbid depression and neurocognitive functions, particularly working memory and cognitive flexibility. However, the small number of studies, moderate heterogeneity, and methodological diversity highlight the need for further research. C-bnt is a potentially promising, cost-effective treatment that warrants exploration as an adjunctive therapy for PTSD.

## 1 Introduction

Post-traumatic stress disorder (PTSD) is a mental disorder that can develop after exposure to traumatic events, defined as actual or threatened death, serious injury, or sexual violence. These events can occur directly, indirectly, or by witnessing them (American Psychiatric Association et al., 2015). Key symptoms of PTSD include re-experiencing the trauma, avoidance behaviors, negative changes in thoughts and mood, and alterations in arousal and reactivity (American Psychiatric Association et al., 2015). In the U. S., the lifetime prevalence of PTSD ranges from 3.4 to 26.9% in civilians and from 7.7 to 17.0% in military populations (Schein et al., 2021). A dissociative subtype was established in DMS-5 (Brewin et al., 2025). This subtype includes symptoms of depersonalization and derealization and is often associated with stronger and more complex symptom severity (Jarkas et al., 2025; Wolf et al., 2016). These differences to PTSD are also reflected in altered neurobiological activation and connectivity patterns (Jarkas et al., 2025). While PTSD is characterized by emotional undermodulation, the dissociative subtype is characterized by emotional overmodulation (Nicholson et al., 2017). In addition, treatment success can be more difficult (Jarkas et al., 2025). Studies suggest that the subtype is common in veterans and people who have experienced child abuse or neglect (Jarkas et al., 2025). In ICD-11, complex PTSD (C-PTSD) was added as a distinct diagnosis (Organization WH, 2022). In addition to the three symptom clusters of PTSD, it includes symptoms that can be summarized as “disturbance in self-organization,” i.e., impairments in affect regulation, a negative self-concept, and interpersonal disturbances (Brewin et al., 2025). Affected individuals have experienced multiple traumatic events or have been exposed to a traumatic event repeatedly or over a prolonged period (Organization WH, 2022). According to studies, patients with C-PTSD report more functional limitations (Brewin et al., 2025).

According to the national S3 guideline of the German Society of Psychotraumatology (DeGPT) for the treatment of PTSD, trauma-focused psychotherapy like Cognitive Behavioral Therapy with trauma focus (CBT-T) and Eye Movement Desensitization and Reprocessing Therapy (EMDR) are the recommended measures for evidence-based treatment (Schäfer et al., 2019). This is in line with results of a recent meta-analysis (Lewis et al., 2020). Psychopharmacotherapy should not be used as first-line treatment (Schäfer et al., 2019), as medications such as fluoxetine, paroxetine, and venlafaxine have demonstrated only small effects (Hoskins et al., 2015).

Cognitive deficits are commonly associated with PTSD, but are often not addressed in trauma therapy (Aupperle et al., 2012; Polak et al., 2012), although is also a predictor of poorer treatment outcome (Fonzo et al., 2019). Psychological trauma can lead to severe and persistent impairments in prospective and retrospective memory, as well as in executive functions (EF) (Polak et al., 2012; Glienke et al., 2017). EF compromises three main cognitive processes: Cognitive flexibility (CF) (the ability to switch between different tasks), working memory (WM), including its limited working memory capacity (WMC) (the ability to store and manipulate relevant information), and inhibition (the ability to control non-goal-directed impulses) (Constantinidis and Klingberg, 2016; Kim et al., 2017).

Research suggests a strong link between the neurobiological basis of cognitive impairments and PTSD core symptoms (Aupperle et al., 2012). PTSD is associated with morphological and functional changes in specific brain regions, particularly the hippocampus, amygdala, and prefrontal cortex (Patel et al., 2012). These regions are known to be involved in memory and higher cognitive functions (Glienke et al., 2017). Cognitive impairments may contribute to the development and persistence of PTSD symptoms, and thus represents an important aspect of effective treatment (Popescu et al., 2023).

Post-traumatic stress disorder is associated with a high risk of comorbid disorders such as depression and anxiety (Frommberger et al., 2014; Illes and Uhl, 2016; Hoppen and Morina, 2019). According to the DSM-5, depression is characterized by persistent depressed mood, loss of interest or pleasure in most activities and fatigue. Other symptoms include cognitive deficits (e.g., lack of attention and concentration) and psychomotor agitation or retardation (American Psychiatric Association et al., 2015). Some evidence suggests that the EF impairments in PTSD may be partly explained by comorbid depressive symptoms, which could act as a mediator for neurocognitive deficits (Olff et al., 2014). However, it is unclear whether the co-occurring depression is an independent disorder or whether it could be a marker that indicates the severity of the symptoms of PTSD (Post et al., 2011). Previous research suggests that military personnel, as compared to civilian samples, are at increased risk of developing depression and PTSD (Rytwinski et al., 2013).

Meta-analyses have confirmed the effectiveness of computer-based cognitive training in improving both depressive symptoms (reduction in symptom severity) and cognitive functions (enhancement in WM, attention and general cognitive performance) (Motter et al., 2016).

### 1.1 Research question and hypotheses

While evidence-based treatments such as CPT-T or prolonged exposure therapy are effective in reducing symptoms in many patients, 20–50% of treated patients with PTSD do not show complete remission of symptoms (Bomyea et al., 2015; Clausen et al., 2019). Study results indicate that computer-based neurocognitive training (c-bnt) can be a promising and feasible option to support psychotherapy (Bomyea et al., 2015; Clausen et al., 2019).

In addition to improving executive functioning, c-bnt may reduce symptoms of PTSD and depression (Bomyea et al., 2015; Clausen et al., 2019; Badura-Brack et al., 2015; Echiverri-Cohen et al., 2021). Impaired cognitive flexibility (CF), attentional switching, and response inhibition are often experienced by patients with PTSD. The literature shows a connection between these impairments and difficulty detaching from salient stimuli experienced by patients with PTSD (Ben-Zion et al., 2018). Training CF, attentional switching and response inhibition can lead to the activation of neuronal plasticity, which can be associated with a functional and behavioral change (Ben-Zion et al., 2018). Bomyea, Stein (Bomyea et al., 2015) demonstrated a reduction in re-experiencing symptoms after c-bnt. Studies suggest that the persistence of intrusions and re-experiencing is due to ineffective use of executive functions. Inhibition training can lead to an improvement in WMC and thus also to a reduction in symptoms (Bomyea et al., 2015). Impairments in response inhibition have been identified as a predictor of poorer symptom remission in patients with post-traumatic stress (Echiverri-Cohen et al., 2021). Studies suggest that impaired response inhibition represents a general deficit in PTSD, contributing to dysfunctional threat processing and the maintenance of symptoms (Aupperle et al., 2012; Echiverri-Cohen et al., 2021). A targeted inhibition-based c-bnt can cause both neural and behavioral alterations. In addition to improving response inhibition, it may also contribute to symptom reduction (Echiverri-Cohen et al., 2021). Although studies have shown c-bnt training to be a promising adjuvant therapy, only a few have investigated its effectiveness for patients with PTSD. The results and designs of these studies are heterogeneous. This meta-analysis and systematic review aim to present and examine the state of the art systematically. The objective of this meta-analysis and review was to investigate whether computer-based neurocognitive training (c-bnt) can improve the severity of PTSD symptoms, comorbid depression and associated deficits in EFs. This led to the following three hypotheses:

1. c-bnt reduces the severity of PTSD symptoms (total score),
2. c-bnt alleviates depressive symptoms (total score),
3. c-bnt enhances CF, WMC, and inhibition.

## 2 Methods

### 2.1 Eligibility criteria and search procedure

For this systematic review and meta-analysis, studies had to be published in English in a peer-reviewed journal and focus on adults (aged > 18 years), whether civilians or military servicemember, affected by PTSD and other deployment-related psychiatric disorders such as depression or anxiety disorder. Inclusion criteria required studies to be RCTs and to involve c-bnt. The search was conducted across the databases PsycINFO, PubMed, Scopus, and PubPsych and was conducted from February 1st, 2023, to June 30st, 2025. Additionally, cross-references from selected studies were reviewed. The electronic search term was: TI = [(PTSD OR “posttraumatic stress disorder” OR “post-traumatic stress disorder” OR “combat PTSD”) AND (attention OR learning OR memory OR inhibition OR cognition OR “computer-based”) AND (intervention OR training)]. The inclusion and search strategy were guided by the PICOS framework: Population – adults diagnosed with PTSD (with or without comorbid depression); Intervention – computer-based neurocognitive training; Comparison – control groups such as waitlist, placebo or active control conditions; Outcomes – PTSD symptom severity, depressive symptoms, executive functions (e.g., cognitive flexibility, working memory, inhibition); Study design –RCTs.

### 2.2 Study selection

Two reviewers screened studies for eligibility, with disagreements resolved by a third reviewer whose decision was final. Records of all screened studies, including reasons for inclusion and exclusion, were maintained. Cross-references from selected studies were also reviewed by the same two reviewers to identify additional studies. All duplicate records identified through the database search were reviewed and removed manually by visual inspection of titles, authors, and publication details.

### 2.3 Data collection and analysis

Selected studies were randomly assigned to three researchers for detailed analysis. Data was systematically extracted, including information on subjects, diagnoses, and neurocognitive assessments, types of c-bnt, and recorded measures. Data availability was checked, and for studies with relevant missing data, the authors were contacted.

### 2.4 Risk of bias

The risk of bias for all included studies was assessed using the revised Cochrane Risk-of-Bias Tool for Randomized Trials (RoB2) (Sterne et al., 2019). This tool evaluates five domains: randomization process, deviations from the intended interventions, missing outcome data, measurement of the outcomes, and selection of reported results. Each study’s overall risk of bias was determined from these domains. Quality assessment was performed by two reviewers, with disagreements resolved by a third reviewer.

### 2.5 Publication bias

Given the small number of studies included in the meta-analyses (*n* = 5), we did not apply formal statistical tests for publication bias such as the Egger test, as such methods are not recommended when fewer than 10 studies are available due to low statistical (Sterne et al., 2011).

### 2.6 Summary of measures and synthesis of results

We conducted two separate meta-analyses: Analysis 1 examined the effect of c-bnt on PTSD symptom severity, and Analysis 2 focused on its effect on depressive symptoms. A third outcome domain, executive functioning, was analyzed descriptively as part of a systematic review, as the available data did not allow for meta-analytic pooling. Sample sizes, mean values, and standard deviations were extracted to calculate Cohen’s *d* effect sizes (Cohen, 1988). Statistical analyses were conducted using IBM SPSS Statistics 29 (IBM Corp. Armonk, NY), applying random effects models, restricted maximum likelihood estimations (REML), and Knapp-Hartung standard error adjustments. Heterogeneity of effect sizes was assessed using *Q*-tests and *I*^2^ statistics, with the following interpretation: low (~25%), moderate (~50%), and high (~75%) (Higgins et al., 2003).

### 2.7 Registration

The meta-analysis and systematic review were registered with PROSPERO (https://www.crd.york.ac.uk/prospero/, registration-ID: CRD42023444417). The Prisma guidelines were used to conduct the meta-analysis and systematic review.

## 3 Results

### 3.1 Study selection

**Figure 1:**
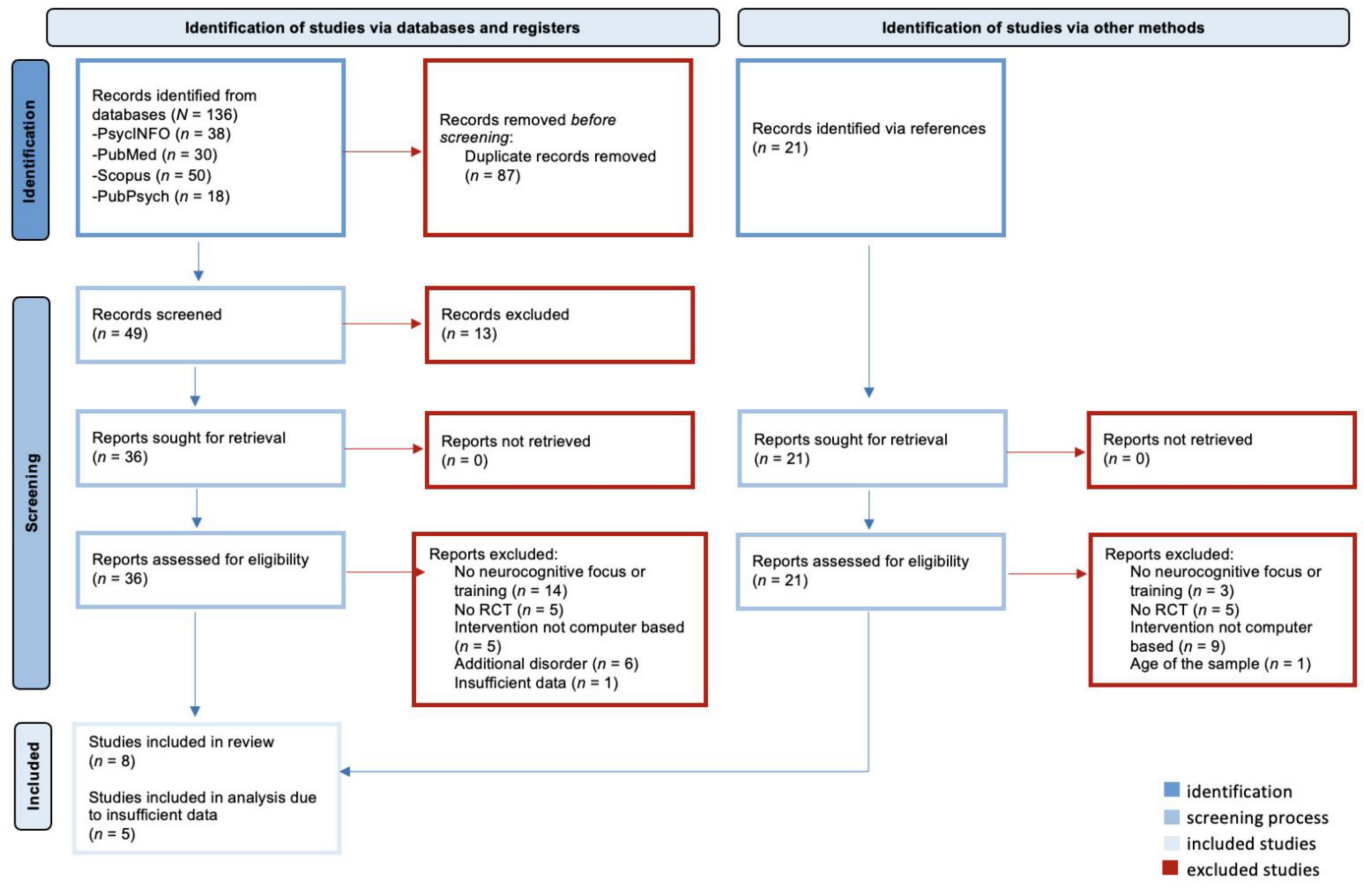
Prisma flow diagram.

The PRISMA Flow Diagram (Figure 1) illustrates the search and selection process (Page et al., 2021). A total of 136 records were identified through database searches. After removing duplicates, 49 abstracts were screened. Of these, 36 studies were assessed in full, 31 were excluded based on eligibility criteria. Additionally, 21 records were identified through reference lists, but 18 of these also did not meet the eligibility criteria. Consequently, 8 studies were included in the systematic review. Due to insufficient data availability, only five studies were included in the meta-analysis.

### 3.2 Study characteristics

The selected studies included participants from different groups: civilians (Bomyea et al., 2015; Echiverri-Cohen et al., 2021; Ben-Zion et al., 2018), active military servicemembers (Kuckertz et al., 2014) and veterans (Fonzo et al., 2019; Clausen et al., 2019; Larsen et al., 2019; Bomyea et al., 2025). All included studies were RCTs. The neurocognitive training interventions varied across all studies. Detailed characteristics of the studies are summarized in Table 1.

**Table 1:** Study characteristics.

Study	Participants	PTSD scale	Depression scale	Executive functioning scale	Training program; length and frequency	Control condition	Main results
Ben-Zion et al. (2018)	Civilians	CAPS	-	Trail Making Test-B Go/No-Go-Task	Executive function training (Lumosity^TM^) and emotional bias training (MyBrainSolutions); 30 days with daily 30 min sessions	Game tasks Reading task	No significant reduction in PTSD symptoms; Significant improvement in CF
Bomyea et al. (2015)	Civilians	CAPS	BDI-II	OSPAN	High interference control training; 4 weeks with two 30 min sessions a week	Low interference control training	Significant reduction on symptoms of re-experience (PTSD) and depressive symptoms; Significant improvement WMC
Bomyea et al. (2025)	Veterans	CAPS-5 PCL-5	-	-	High interference control training; 8 weeks with two 30 min sessions a week	Low interference control training	No significant reduction in PTSD symptoms
Clausen et al. (2019)	Veterans	PCL-M	BDI-II	Trail Making Test-B	Executive function training (Lumosity^TM^); 6 weeks with 30 min sessions five times a week	Placebo training (Puzzles, crosswords, hangman etc.)	Reduction in PTSD and depressive symptoms; No improvement CF
Echiverri-Cohen et al. (2021)	Civilians	PDS	QIDS-SR16	Go/No-Go-Task Stop-Signal-Task	Response Inhibition Training (RIT); 2 days with 3 h sessions a day	Waitlist	Reduction in PTSD symptoms; No reduction in depressive symptoms; No improvement in trained inhibition task, but significant improvement in untrained inhibition transfer task
Fonzo et al. (2019)	Veterans and civilians	PCL CAPS	BDI	Trail Making Test-B Go/No-Go-Task	Game-like tasks: selective attention, working memory, task shifting, processing speed, positive emotion recognition/resisting negative emotion distraction; 45 days with daily sessions of 35–45 min	Computer games that did not train specific cognitive function	No significant reduction in PTSD symptoms; Chances in depressive symptoms not reported; No improvement in CF or inhibition
Kuckertz et al. (2014)	Active soldiers	PCL-M	BDI-II	–	Attention Bias Modification (ABM); 2 weeks with daily sessions (no specified time)	Attention control condition (ACC)	Significant reduction in PTSD and depression symptoms
Larsen et al. (2019)	Veterans	PCL-5	DASS-D	OSPAN RSPAN SSPAN	Emotional working memory training (n-back) 5 weeks with 15 sessions of 20 min	Emotional working memory training (1-back)	Reduction in PTSD symptoms; No significant reduction in depressive symptoms; No improvement in WMC

### 3.3 Risk of bias

The risk of bias was assessed using the Cochrane Risk-of-Bias Tool for Randomized Trials (RoB2) (Higgins et al., 2019). The analysis of the randomization process indicated a low risk of bias in seven studies (Fonzo et al., 2019; Bomyea et al., 2015; Clausen et al., 2019; Ben-Zion et al., 2018; Kuckertz et al., 2014; Larsen et al., 2019; Bomyea et al., 2025). Deviations from intended interventions showed a low risk in five studies (Clausen et al., 2019; Echiverri-Cohen et al., 2021; Ben-Zion et al., 2018; Kuckertz et al., 2014; Bomyea et al., 2025), while Bomyea et al. (2015), Fonzo et al. (2019), and Larsen et al. (2019) raised some concerns at this level. For missing outcome data, Bomyea et al. (2015), Bomyea et al. (2025), Fonzo et al. (2019), Kuckertz et al. (2014), and Larsen et al. (2019) were assessed as having a low risk of bias. However, concerns were noted in the studies by Echiverri-Cohen et al. (2021), Ben-Zion et al. (2018), and Clausen et al. (2019). Regarding outcome measurement, only Fonzo et al. (2019) raised some concerns, while the other seven studies had a low risk.

**Figure 2:**
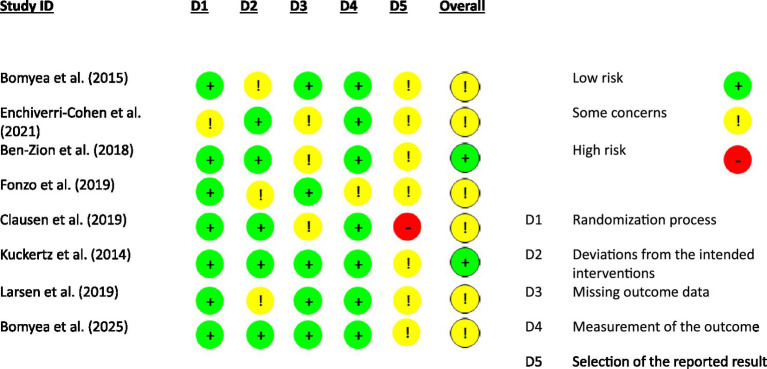
Risk of bias.

In terms of the selection of reported results, Clausen et al. (2019) demonstrated a high risk of bias, whereas the other studies showed some concerns. Overall, the risk of bias analysis concluded that studies by Bomyea et al. (2015), Bomyea et al. (2025), Echiverri-Cohen et al. (2021), Fonzo et al. (2019), Clausen et al. (2019), and Larsen et al. (2019) had some concerns, while Ben-Zion et al. (2018) and Kuckertz et al. (2014) had an overall low risk of bias. An overview of these results is presented in Figure 2.

### 3.4 Publication bias

Due to the inclusion of only 8 studies in our meta-analysis, it was not possible to evaluate publication bias.

### 3.5 Results of individual studies and synthesis of results

#### 3.5.1 PTSD

The studies of Kuckertz et al. (2014), Larsen et al. (2019), Echiverri-Cohen et al. (2021), Bomyea et al. (2015), and Bomyea et al. (2025) were included in Analysis 1, which examined the influence of neurocognitive training on PTSD symptoms (see Table 2 for means and standard deviations). Studies by Ben-Zion et al. (2018), Clausen et al. (2019), and Fonzo et al. (2019) were excluded due to insufficient data.

**Table 2:** Descriptive statistics for PTSD.

	EG			CG		
	*n*	*M*	*SD*	*n*	*M*	*SD*
Bomyea et al. (2015)	22	45.32	19.92	20	58.50	18.61
Echiverri-Cohen et al. (2021)	24	34.38	15.04*	25	37.76	18.90*
Kuckertz et al. (2014)	12	42.83	12.04	17	51.65	14.72
Larsen et al. (2019)	10	40.90	12.11	10	38.00	13.56
Bomyea et al. (2025)	42	28.34	13.60	39	26.29	14.00

EG = experimental group; CG = control group; *n* = sample size; *M* = mean; *SD* = standard deviation. *Calculated from standard error.

**Figure 3:**
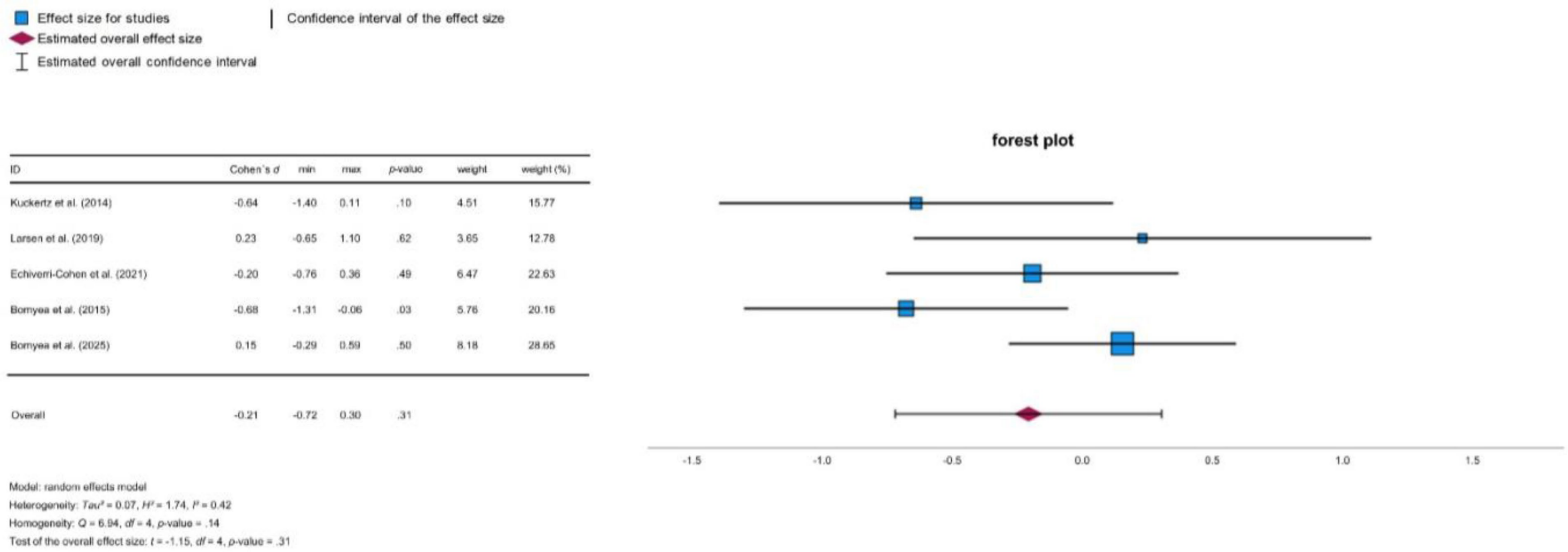
Forest plot PTSD.

All included studies reported improvements in PTSD symptom severity in the training group, although the extent of improvement varied. Ben-Zion et al. (2018) observed a more pronounced benefit for the training group compared to the control group. Bomyea et al. (2015) found that the training group had significantly lower scores on symptoms of re-experiencing, but no differences were reported in other types of PTSD symptoms. Clausen et al. (2019) and Larsen et al. (2019) noted improvements in PTSD symptoms in both groups post-training. Echiverri-Cohen et al. (2021) reported a reduction in PTSD symptoms from pre- to post-training in the training group, while symptoms in the waitlist condition increased. However, group differences in PTSD severity were significant at pre-training but not at post-training (due to unmatched groups), but not at post-training. Kuckertz et al. (2014) found a significant reduction in PTSD symptoms in the training group after completion of the training. Fonzo et al. (2019) did not observe any significant training-related changes, although there was a trend toward improvement, particularly in re-experiencing symptoms. Similarly, Bomyea et al. (2025) observed reductions in PTSD symptoms in both groups, although no significant differences between the training conditions emerged post-treatment. According to Cohen (1988), Analysis 1 revealed a small effect size (*d* = −0.21, 95% CI [−0.72, 0.30]; see Figure 3); however, this effect was not statistically significant (*p* = 0.31), indicating no reliable reduction in PTSD symptoms across studies. The heterogeneity test indicated moderate heterogeneity (*Q* = 6.94, *p* = 0.14, *I*^2^ = 42.4%).

**Figure 4:**
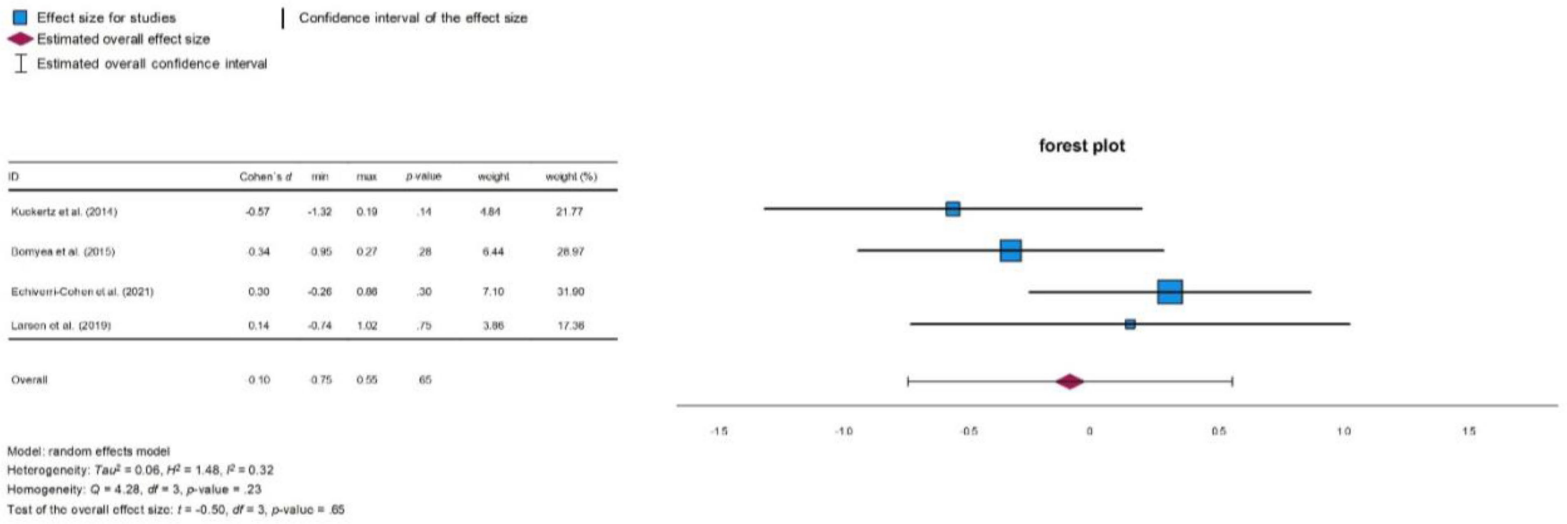
Forest plot depression.

#### 3.5.2 Depression

In Analysis 2, which examined the influence of neurocognitive training on depressive symptoms, the studies Kuckertz et al. (2014), Larsen et al. (2019), Echiverri-Cohen et al. (2021), and Bomyea et al. (2015) were included (for means and standard deviations see Table 3). The results across the studies varied. Some reported improvements in the severity of depressive symptoms in the training group, while others found no significant changes.

**Table 3:** Descriptive statistics for depression.

Study	EG			CG		
	*n*	*M*	*SD*	*n*	*M*	*SD*
Bomyea et al. (2015)	22	20.36	14.76	20	25.05	12.91
Echiverri-Cohen et al. (2021)	24	13.85	6.07*	25	12.03	6.10*
Kuckertz et al. (2014)	12	14.67	9.61	17	22.07	14.90
Larsen et al. (2019)	10	21.33	12.17	10	19.80	9.31

EG = experimental group; CG = control group; *n* = sample size; *M* = mean; *SD* = standard deviation; *calculated from standard error.

Bomyea et al. (2015) observed reductions in depressive symptoms in both training and control groups. Similarly, Echiverri-Cohen et al. (2021) found no significant changes pre- and post-training in either group. On the other hand, Kuckertz et al. (2014) reported a significant reduction in depressive symptoms in the training group following completion of the intervention. In contrast, Larsen et al. (2019) found no significant changes in depressive symptoms in either group. Although Clausen et al. (2019) reported an improvement in depressive symptoms after completion of the training, their data was unavailable for inclusion in our analysis. Fonzo et al. (2019) also assessed depressive symptoms, but did not report the relevant results. Overall, Analysis 2 showed no significant effect of neurocognitive training on depressive symptoms according to Cohen (1988) (*d* = −0.10, 95% CI [−0.75, 0.55]; see Figure 3). The heterogeneity test indicated moderate variability between the studies (*Q* = 4.28, *p* = 0.233, *I*^2^ = 32.4%).

#### 3.5.3 Executive functioning

The studies by Ben-Zion et al. (2018), Bomyea et al. (2015), Clausen et al. (2019), Fonzo et al. (2019), Echiverri-Cohen et al. (2021), and Larsen et al. (2019) were analyzed to address the hypothesis of whether c-bnt improves EF. The primary EF components targeted were CF, inhibition, and (WMC). The Trail Making Test B (TMT-B), Go/No-Go task, and the Operation Span Task (OSPAN) were used as appropriate measures of these domains. Due to inconsistent availability of data across studies, a statistical comparison of the test variables (TMT-B, Go/No-Go task, and OSPAN) for meta-analysis was not feasible. Therefore, the results regarding EF improvements are summarized in a systematic review.

In Ben-Zion et al. (2018), CF (TMT-B) improved in the intervention group following neuropsychological intervention. Clausen et al. (2019) used different neuropsychological tests to calculate two global average scores: executive functioning and neuropsychological functioning. Neither global score, including TMT-B, showed significant improvement.

In Fonzo et al. (2019), results for CF (TMT-B) and inhibition (Go/No-Go task) are not reported in the main findings, and Supplementary data indicated no improvement in either domain after training.

Echiverri-Cohen et al. (2021) assessed response inhibition using two tests, comparing the effects of response inhibition training on both trained and untrained transfer tasks (Go/No-Go task and Stop Signal task). While there was no improvement in the trained modified Go/No-go task, response inhibition in the untrained Stop Signal task improved after training.

Bomyea et al. (2015) reported a significant improvement in WMC capacity (OSPAN) in the intervention group post-training. In contrast, Larsen et al. (2019) found no significant differences in WMC (OSPAN) between the intervention and control group after n-back training.

## 4 Discussion

In this study, we examined the effects of c-bnt as an adjuvant treatment of PTSD in adults. We assessed three key variables: PTSD, depressive symptomatology, and neurocognitive functioning. A total of eight studies were included in the systematic review, with five providing sufficient data for the Meta-Analysis 1 (effects on PTSD symptoms) and Meta-Analysis 2 (effects on depressive symptoms). Six studies were examined regarding their impact on cognitive flexibility, inhibition, and working memory capacity. However, due to incomplete reporting and considerable variation in outcome measures, a meta-analytic synthesis was not feasible. This is the first meta-analysis to examine the efficacy of c-bnt on PTSD with comorbid depression, providing pioneering insights that can guide further research. Notably, this meta-analysis does not only focus on clinical symptoms, but also closely examines the broad spectrum of neurocognitive functions associated with PTSD. While a small to medium reduction in overall PTSD symptom severity was observed in the meta-analysis, the effect was not statistically significant and should therefore be interpreted cautiously. For the first time, we observed a reduction in PTSD symptom severity in patients undergoing c-bnt. In some studies, improvements were also reported in the control groups, which may be attributable to non-specific effects, active control interventions, or concurrent treatments. These findings highlight the need for carefully designed control conditions in future research. Regarding comorbid depressive symptoms, there was a non-significant trend toward symptom improvement, which may indicate potential effects that require confirmation in larger samples. In addition, a systematic review was conducted to explore the efficacy of c-bnt on three neurocognitive domains commonly impaired in in PTSD: CF, inhibition, and WMC. While improvements in CF and WMC were observed following c-bnt, the results were inconsistent due to methodological differences across studies. For inhibition, no significant improvement was found, though a transfer effect was observed.

### 4.1 PTSD

Meta-analysis 1 indicated a small reduction in PTSD symptoms following neurocognitive training, although this effect did not reach statistical significance. While the direction of the effect was not consistently negative across studies, the wide confidence interval and non-significant result do not support a reliable effect of c-bnt on PTSD symptoms at this stage. All eight studies included in the analysis reported a reduction in PTSD symptom severity in the training group, although the magnitude and consistency of effects varied. Some studies showed statistically significant improvements, while others only revealed trends or no group differences. This finding is notable given the substantial variability in subjects, psychotherapeutic treatments, and neurocognitive training protocols across the reviewed studies. Subjects were active military servicemembers, veterans, civilians, with male or female gender and with different types of trauma, ranging from sexual trauma to combat trauma. These differences likely influence the severity of PTSD symptoms and the effectiveness on trauma treatments. Research indicates that sexual trauma is associated with greater symptom severity compared to other trauma types (Jakob et al., 2017). Combat trauma is often linked with increased re-experiencing and hyperarousal, whereas sexual trauma is more closely associated with avoidance and negative cognitions (Guina et al., 2018).

Another potentially influential factor is the state of PTSD chronification. Only two studies provided information on whether the PTSD was acute or chronic (Fonzo et al., 2019; Ben-Zion et al., 2018), leaving the chronification status unclear in the remaining studies. This variability makes it difficult to rule out a heterogeneous distribution. According to McGorry et al. (2006), early interventions may have a better prognosis regarding effectiveness and progression in PTSD treatment. Optimizing neurocognitive training to address the specific needs of different patient groups could potentially enhance its effectiveness.

Some studies included in this review also involved heterogeneous psychopharmacological treatments and/or psychotherapy parallel with c-bnt. Kuckertz et al. (2014) and Larsen et al. (2019) administered concurrent psychological treatment and pharmacotherapy in both the experimental and control groups, leading to significant improvements in PTSD symptoms (PCL score) in both groups. However, both conditions in these studies were highly similar. Larsen et al. (2019) who report that both groups improved significantly, stated themselves that the control condition was equivalent to an intervention, and study participants of both the experimental and the control group subjectively judged the tasks as challenging. In contrast, Echiverri-Cohen et al. (2021) used a waiting group as a control, where PTSD symptoms increased compared to the training group, which showed a decrease in symptoms from pre- to post-training. Similarly, Bomyea et al. (2025) found reductions in PTSD symptoms in both training groups, but no significant differences between high and low interference conditions at post-treatment. To better assess the effects attributable to neurocognitive training, more distinct control conditions are needed. Previous meta-analyses have not yet demonstrated improved outcomes for PTSD patients through the combination of psychotherapy and pharmacotherapy (Hetrick et al., 2010). Future studies with larger samples sizes and more homogeneous patient groups - regarding trauma, chronification and comorbid disorders–are necessary. We assume that well conceptualized c-bnt, when combined with psychotherapy and individually tailored pharmacotherapy, could lead to symptom improvement. For example, noradrenergic antidepressants might improve sleep in patients with comorbid insomnia, thereby improving cognitive resources needed to benefit from combined neurocognitive training and trauma therapy.

C-bnt programs varied significantly in content, duration, and targeted cognitive functions. Larsen et al. (2019), Clausen et al. (2019), Fonzo et al. (2019), Ben-Zion et al. (2018), and Bomyea et al. (2015) focused on WM. Inhibition capacities were targeted by Echiverri-Cohen et al. (2021), Clausen et al. (2019), and Ben-Zion et al. (2018), while attention was the focus of Fonzo et al. (2019), and Kuckertz et al. (2014). In addition, the duration of training sessions varied widely, from as short as 2 days (Echiverri-Cohen et al., 2021) to as long as 6 weeks (Clausen et al., 2019), with session lengths ranging from 20 min (Larsen et al., 2019) to 3 h (Echiverri-Cohen et al., 2021), and frequencies from twice a week (Bomyea et al., 2015) to daily (Kuckertz et al., 2014).

Lampit et al. (2014) demonstrated in their meta-analysis that training sessions conducted more than three times a week did not show significant effects. They also indicated that computer-based cognitive training sessions should last longer than 30 min to be effective. We agree with the Lampit, Hallock’s (Page et al., 2021) main findings and suggest that extending the duration of c-bnt could potentially yield more sustainable results. However, the stability of training effects and the transferability of trained cognitive tasks to untrained cognitive everyday challenges still remain a matter of debate. Further research and long-term studies with follow-ups are needed to gain more insights into these central issues.

Studies that integrated affective and cognitive elements into their training programs have demonstrated a significant reduction in symptoms in pre-post study designs (Kuckertz et al., 2014; Larsen et al., 2019). Larsen et al. (2019) trained with an emotional n-back task, while Kuckertz et al. (2014) used trauma-triggering words in their attentional bias modification (ABM). The effects combining cognitive and affective elements warrant further investigations. Future research could benefit from including both a control group and two experimental groups: one receiving solely cognitive training and the other receiving training that integrates both cognitive and affective components. Although the studies are heterogeneous in terms of the above-discussed points, our updated meta-analysis indicates moderate statistical heterogeneity of training effects on PTSD. These findings reflect inconsistent results across studies and suggest that the effectiveness of c-bnt in reducing PTSD symptoms remains uncertain. Given the ease of implementation, flexibility, cost-effectiveness, and the potential positive impact of c-bnt, we recommend further investigating c-bnt as a potential complementary approach in the treatment of PTSD.

### 4.2 Depression

Meta-analysis 2 found that neurocognitive training had no statistically significant effect on comorbid depressive symptoms in patients with PTSD, but there was a tendency toward improvement (Bomyea et al., 2015; Echiverri-Cohen et al., 2021; Kuckertz et al., 2014; Larsen et al., 2019).

Rock et al. (2014), in their meta-analysis, reported that individuals with depression exhibit moderate cognitive deficits in EF and attention, and these deficits often persist even after the depressive symptoms subside. This emphasizes the need for neurocognitive training in depressive patients. Although our meta-analysis did not focus on depression as a primary disorder, our findings align with this argument. Prior meta-analyses specifically targeting depression as a primary disorder have shown reductions in depressive symptoms following computerized cognitive training (Motter et al., 2016). However, we could not replicate these findings. A potential explanation is that our primary target disorder was PTSD, and depression was examined in only a subset of studies where it was a comorbid condition.

Rosen et al. (2020) reported that 80% of PTSD cases occur with comorbid disorders, with depression present in 50% of cases. Our meta-analysis addresses this issue by showing depression was a comorbid condition in 86% of the reviewed studies. Given this high comorbidity, our findings suggest that neurocognitive training may have the potential to improve symptoms of both PTSD and depression. Previous meta-analyses, such as Motter et al. (2016), reported that computerized cognitive training (CCT) can improve both depressive symptoms and cognitive functioning. More recently, Launder et al. conducted a comprehensive meta-analysis of randomized controlled trials and confirmed moderate effects of CCT on overall depressive symptomatology, working memory, and attention. These findings highlight the potential of CCT approaches, although our meta-analysis did not replicate these effects in patients with PTSD and comorbid depression.

### 4.3 Executive functioning

#### 4.3.1 Cognitive flexibility

The evidence regarding the efficacy of c-bnt on CF is heterogeneous. While Ben-Zion et al. (2018) identified improvements in CF (TMT-B), Clausen et al. (2019), and Fonzo et al. (2019) did not observe any changes. Methodological differences may at least in part explain these contradicting results. For instance, Clausen et al. (2019) created two global scores by averaging results from various neuropsychological tests, including TMT-B. This approach limits the ability to analyze individual test results in detail. Moreover, differences in the training programs could contribute to these divergent findings. Ben-Zion et al. (2018) and Clausen et al. (2019) used the same c-bnt, they focused on response inhibition, attention and task shifting, WM, and processing speed. Fonzo et al. (2019) used a different training program and focused on similar domains, including shared attention, working memory, and task shifting. Notably, Ben-Zion et al. (2018) and Fonzo et al. (2019) extended their cognitive training with tasks that included an affective component. This combination makes it difficult to differentiate between cognitive and emotional training effects. Emotional stimuli are particularly salient in individuals with PTSD (Hayes et al., 2012). Furthermore, studies have postulated that enhanced activation of the neural fear network may reduce the capacity for processing non-threatening information (Hayes et al., 2012). Therefore, the question needs to be clarified, which kind of emotional stimuli and tasks can be efficiently applied in c-bnt in patients with PTSD. To assess the effectiveness of a c-bnt and to manage the impact of emotional stimuli, it would be useful to implement a tailored training program that focuses on specific functions. This may comprise either a cognitive or an affective training component.

Differences in training duration likely also contribute to the heterogeneous outcomes of c-bnt. Training periods varied between 30 days (Ben-Zion et al., 2018) with 30 min per day, 42 days with 30 min per day (Clausen et al., 2019), and 45 days with 35–45 min per day (Fonzo et al., 2019). Samples differ between these three studies, which may also affect results. Ben-Zion et al. (2018) included civilians, Clausen et al. (2019) focused combat veterans, and Fonzo et al. (2019) studied both veterans and civilians. Treatment efficacy observed in military samples is lower than that observed in civil samples (Williamson et al., 2023). It is conceivable that neurocognitive training may have a more pronounced impact on civilian samples. Additionally, hypervigilance is often associated with combat trauma (Kimble et al., 2013). The constant scanning of the environment for threatening stimuli might negatively affect CF. It is possible that available cognitive training programs are not well suited for soldiers, and that a distinct type of training program may be required for military personnel. To better understand the variables influencing the effectiveness of c-bnt, examining civilian and military personnel separately could be valuable.

#### 4.3.2 Inhibition

In the reviewed studies, cognitive training did not yield a significant improvement in inhibition as measured by the Go/No-Go task (Fonzo et al., 2019; Echiverri-Cohen et al., 2021). However, Echiverri-Cohen et al. (2021) reported a transfer effect. They used a response inhibition training (RIT), a c-bnt that applies randomly assigned visual stimuli (letters and colors) for a Go-No-Go task. Inhibition was assessed before and after training using two tests: a modified version of the trained task (modified Go/No-Go task) and an untrained transfer task (Stop Signal task). While no differences were observed in the trained task, there was a significant improvement in the untrained transfer task. During training, colors and letters were used as Go stimuli. At the pre- and post-assessments, a modified Go/No-Go task with symbols was used. It is possible that the alteration between the stimuli (colors and letters to symbols) influenced performance. Attention to the trained Go stimuli may have been increased and thus the switch to symbols may have had a negative effect on performance. This argument, however, does not explain the significant improvement in the untrained Stop Signal task. It is possible that the training has initiated a process of synaptic plasticity, which has resulted in enhanced performance on the transfer task (Shipstead et al., 2012). C-bnt should target the transfer effect and improve the underlying ability (Shipstead et al., 2012). Accordingly, the results of Echiverri-Cohen et al. (2021) are highly interesting. Transfer mechanisms could well to be assessed in combined neuropsychological and functional neuroimaging studies with pre-post training experimental designs. In this context, it is also important to note that in the study of Echiverri-Cohen et al. (2021), civilians completed two individual training sessions of approximately 3 h. In Fonzo et al. (2019), civilians and veterans engaged in training for 45 days, with daily sessions lasting 35–45 min. The results of Echiverri-Cohen et al. (2021) suggest that brief, individualized training may lead to improved performance in a transfer task in civilian, and perhaps also military samples.

#### 4.3.3 Working memory capacity

Results regarding the efficacy of c-bnt on WMC in patients with PTSD are also heterogeneous. Bomyea et al. (2015) reported a significant improvement in WMC (OSPAN), while Larsen et al. (2019) found no significant differences after c-bnt. The contrasting results can at least in part be explained by the different training programs used. Bomyea et al. (2015) applied a modified reading span WM task. The training conducted by Larsen et al. (2019) involved an emotional n-back task. They distinguished between two modalities: visually presented faces with fearful expressions and auditory presented negative words. It is possible that the use of emotionally negative stimuli in the context of WMC is not an effective approach. As stated above in paragraph on cognitive flexibility, the increase in the neural fear network following PTSD may have compromised WMC (Hayes et al., 2012). The results of Bomyea et al. (2015) demonstrate that the use of neutral stimuli is effective in improving WMC.

Differences in training intensity between the studies may also contribute to the observed heterogeneity: participants in Bomyea et al. (2015) completed eight 30-min training sessions over 4 weeks, whereas subjects in Larsen et al. (2019) participated in 15 20-min sessions over 5 weeks. This suggest that the effectiveness of a c-bnt on the WMC may be more related to the duration of training sessions rather than their frequency.

Additionally, the results may be influenced by factors such as sample characteristics and the type of trauma. Bomyea et al. (2015) included civilian women after sexual trauma, whereas Larsen et al. (2019) recruited both female and male veterans. Combat trauma often causes re-experiencing symptoms, associated with WM deficits (Guina et al., 2018). It is possible that combat-related trauma is associated with greater deficits in WM, which may make it more challenging to receive effective WM training for soldiers.

Studies show that WMC can be increased by training, the exact mechanisms of action in PTSD need to be further investigated (Constantinidis and Klingberg, 2016).

### 4.4 Limitations

This review is subject to several limitations. First, only a small number of studies met inclusion criteria, and just five provided sufficient data for meta-analyses, which limits statistical power and generalizability. Due to inconsistent reporting and heterogeneity in cognitive tasks, no meta-analysis could be conducted for executive functions. Moreover, no follow-up assessments were reported, leaving the long-term effectiveness of c-bnt unclear.

Second, while a random-effects model with REML estimation and Knapp-Hartung adjustment was used, methods recommended for small samples, this approach may yield conservative estimates, particularly in contexts of moderate heterogeneity. In the present analysis, heterogeneity was higher than previously reported (*I*^2^ = 42.4% vs. *I*^2^ = 7.1%), suggesting that the included studies differ more substantially regarding their populations, interventions, and outcomes than initially assumed. Effect sizes were calculated using Cohen’s d, which may slightly overestimate effects in small samples. Future meta-analyses should consider using alternative estimators such as Hedges’ *g*.

Third, subgroup or moderator analyses (e.g., military versus civilian populations, trauma type, PTSD chronicity, or comorbidity profiles) were not conducted, despite known differences in symptomatology and treatment response. Dropout rates and adherence data were also not systematically reported or analyzed, limiting conclusions regarding intervention feasibility and participant engagement.

Fourth, the substantial heterogeneity of training protocols (e.g., targeted cognitive domains, emotional content, session frequency and duration) was not formally categorized, which hinders identification of the most effective intervention components. The newly added study by Bomyea et al. (2025) further increased variability by introducing a larger Veteran sample and an extended training protocol. Additionally, due to the limited number of studies, no leave-one-out sensitivity analysis was performed, as such procedures can yield unstable or misleading results in small datasets. Similarly, no formal assessment of publication bias was conducted, as standard methods (e.g., Egger’s test, Trim-and-Fill) are considered unreliable when fewer than 10 studies are available.

Lastly, the included studies varied in terms of intervention content, outcome measures, and sample characteristics, which may have introduced further heterogeneity and limited comparability. This diversity, combined with the small sample sizes and the lack of significant overall effects, underscores the need for further well-powered RCTs to clarify the role of c-bnt in PTSD treatment.

## 5 Conclusion

C-bnt yielded a small, non-significant effect on PTSD symptom severity; however, this effect did not reach statistical significance and should be interpreted with caution. Compared to previous analyses, the updated results confirm a small and inconclusive effect with moderate heterogeneity across studies. Despite the moderate statistical heterogeneity, the current evidence remains inconclusive regarding its clinical efficacy.

Additionally, there was a tendency toward improvement in comorbid depressive symptoms, underscoring the importance of considering comorbid depression in PTSD treatment approaches. C-bnt led to improvements in CF and WM in civilian samples, and a transfer effect was observed in inhibition, suggesting that c-bnt may enhance training-specific performance and potentially promote improvements in skills relevant in daily life. However, the review highlighted discrepancies in the efficacy of c-bnt between civilian and military populations, indicating that sample- and trauma-specific effect mechanisms of c-bnt need further investigations.

Future studies should focus on larger and more homogeneous samples, use standardized outcome measures (CAPS-5 as primary clinical endpoint), and clearly differentiate between intervention effects and non-specific treatment factors. In addition, research should examine the long-term sustainability of c-bnt effects and their integration into trauma-focused psychotherapy.

We emphasize the importance of understanding the data and calculations involved to fully harness the potential of studies like meta-analyses. For this reason, we advocate for transparency, data traceability, and publication of raw data. The CONSORT (Consolidated Standards of Reporting Trials) statement proposed by Moher, Hopewell (Moher et al., 2010) offers valuable guidelines for improving reporting standards. Furthermore, the concept of EFs is applied inconsistently across studies. Therefore, precise operational definitions and standardized assessments are necessary for advancing research in this area.

## Data Availability

The raw data supporting the conclusions of this article will be made available by the authors, without undue reservation.

## References

[ref1] American Psychiatric Association Falkai P. Wittchen H. Döpfner M. (2015). Diagnostisches und Statistisches Manual Psychischer Störungen DSM-5®. Washington, DC: Hogrefe.

[ref2] Aupperle R. L. Melrose A. J. Stein M. B. Paulus M. P. (2012). Executive function and PTSD: disengaging from trauma. Neuropharmacology 62, 686–694. doi: 10.1016/j.neuropharm.2011.02.008, PMID: 21349277 PMC4719148

[ref3] Badura-Brack A. S. Naim R. Ryan T. J. Levy O. Abend R. Khanna M. M. . (2015). Effect of attention training on attention bias variability and PTSD symptoms: randomized controlled trials in Israeli and US combat veterans. Am. J. Psychiatry 172, 1233–1241. doi: 10.1176/appi.ajp.2015.14121578, PMID: 26206075 PMC6343502

[ref4] Ben-Zion Z. Fine N. B. Keynan N. J. Admon R. Green N. Halevi M. . (2018). Cognitive flexibility predicts PTSD symptoms: observational and interventional studies. Front. Psych. 9:477. doi: 10.3389/fpsyt.2018.00477, PMID: 30337890 PMC6180246

[ref5] Bomyea J. Caudle M. Bartolovich A. Simmons A. Jak A. Golshan S. (2025). Randomized controlled trial of computerized working memory training for veterans with PTSD. J. Psychiatr. Res. 181, 350–357. doi: 10.1016/j.jpsychires.2024.11.072, PMID: 39642474 PMC12425496

[ref6] Bomyea J. Stein M. B. Lang A. J. (2015). Interference control training for PTSD: a randomized controlled trial of a novel computer-based intervention. J. Anxiety Disord. 34, 33–42. doi: 10.1016/j.janxdis.2015.05.010, PMID: 26114901 PMC4532583

[ref7] Brewin C. R. Atwoli L. Bisson J. I. Galea S. Koenen K. Lewis-Fernández R. (2025). Post-traumatic stress disorder: evolving conceptualization and evidence, and future research directions. World Psychiatry 24, 52–80. doi: 10.1002/wps.21269, PMID: 39810662 PMC11733483

[ref8] Clausen A. N. Thelen J. Francisco A. J. Bruce J. Martin L. McDowd J. . (2019). Computer-based executive function training for combat veterans with PTSD: a pilot clinical trial assessing feasibility and predictors of dropout. Front. Psych. 10:62. doi: 10.3389/fpsyt.2019.00062, PMID: 30881315 PMC6405637

[ref9] Cohen J. (1988). Statistical power analysis for the Behavioral sciences. Hillsdale, NJ: Lawrence Erlbaum Associates.

[ref10] Constantinidis C. Klingberg T. (2016). The neuroscience of working memory capacity and training. Nat. Rev. Neurosci. 17, 438–449. doi: 10.1038/nrn.2016.43, PMID: 27225070

[ref11] Echiverri-Cohen A. Spierer L. Perez M. Kulon M. Ellis M. D. Craske M. (2021). Randomized-controlled trial of response inhibition training for individuals with PTSD and impaired response inhibition. Behav. Res. Ther. 143:103885. doi: 10.1016/j.brat.2021.103885, PMID: 34089923

[ref12] Fonzo G. A. Fine N. B. Wright R. N. Achituv M. Zaiko Y. V. Merin O. . (2019). Internet-delivered computerized cognitive & affective remediation training for the treatment of acute and chronic posttraumatic stress disorder: two randomized clinical trials. J. Psychiatr. Res. 115, 82–89. doi: 10.1016/j.jpsychires.2019.05.007, PMID: 31125916

[ref13] Frommberger U. Angenendt J. Berger M. (2014). Post-traumatic stress disorder—a diagnostic and therapeutic challenge. Dtsch. Arztebl. Int. 111, 59–65. doi: 10.3238/arztebl.2014.0059, PMID: 24612528 PMC3952004

[ref14] Glienke K. Willmund G.-D. Zimmermann P. Piefke M. (2017). Complex real life-related prospective memory in soldiers with and without post-traumatic stress disorder. J Trauma Stress Disord Treat. 6, 171–181. doi: 10.4172/2324-8947.1000176, PMID: 39887974

[ref15] Guina J. Nahhas R. W. Sutton P. Farnsworth S. (2018). The influence of trauma type and timing on PTSD symptoms. J. Nerv. Ment. Dis. 206, 72–76. doi: 10.1097/NMD.0000000000000730, PMID: 29271827

[ref16] Hayes J. P. Vanelzakker M. B. Shin L. M. (2012). Emotion and cognition interactions in PTSD: a review of neurocognitive and neuroimaging studies. Front. Integr. Neurosci. 6:89. doi: 10.3389/fnint.2012.00089, PMID: 23087624 PMC3466464

[ref17] Hetrick S. E. Purcell R. Garner B. Parslow R. (2010). Combined pharmacotherapy and psychological therapies for post traumatic stress disorder (PTSD). Cochrane DB Syst Rev. 7:CD007316. doi: 10.1002/14651858.CD007316.pub2, PMID: 20614457 PMC12515543

[ref18] Higgins J. P. Savović J. Page M. J. Sterne J. A. (2019). “Revised Cochrane Risk-of-Bias Tool (RoB 2)” in Cochrane handbook for systematic reviews of interventions.

[ref19] Higgins J. P. Thompson S. G. Deeks J. J. Altman D. G. (2003). Measuring inconsistency in meta-analyses. BMJ 327, 557–560. doi: 10.1136/bmj.327.7414.557, PMID: 12958120 PMC192859

[ref20] Hoppen T. H. Morina N. (2019). The prevalence of PTSD and major depression in the global population of adult war survivors: a meta-analytically informed estimate in absolute numbers. Eur. J. Psychotraumatol. 10:1578637. doi: 10.1080/20008198.2019.1578637, PMID: 30834069 PMC6394282

[ref21] Hoskins M. Pearce J. Bethell A. Dankova L. Barbui C. Tol W. A. . (2015). Pharmacotherapy for post-traumatic stress disorder: systematic review and meta-analysis. Br. J. Psychiatry 206, 93–100. doi: 10.1192/bjp.bp.114.148551, PMID: 25644881

[ref22] Illes F. Uhl I. (2016). Posttraumatische Belastungsstörung und Depression. Nervenheilkunde 35, 474–480. doi: 10.1055/s-0037-1616408, PMID: 40738129

[ref23] Jakob J. M. Lamp K. Rauch S. A. Smith E. R. Buchholz K. R. (2017). The impact of trauma type or number of traumatic events on PTSD diagnosis and symptom severity in treatment seeking veterans. J. Nerv. Ment. Dis. 205, 83–86. doi: 10.1097/NMD.0000000000000581, PMID: 28129258

[ref24] Jarkas D. A. Robillard R. Malenfant C.-R. Richards C. Lanthier M. Beaurepaire C. . (2025). Exploring the dissociative subtype of PTSD: the role of early-life trauma, cortisol, and inflammatory profiles. Psychoneuroendocrinology 175:107406. doi: 10.1016/j.psyneuen.2025.107406, PMID: 40010078

[ref25] Kim N. Y. Wittenberg E. Nam C. S. (2017). Behavioral and neural correlates of executiver function: interplay between inhibition and updating processes. Front. Neurosci. 11:378. doi: 10.3389/fnins.2017.00378, PMID: 28713237 PMC5492464

[ref26] Kimble M. O. Fleming K. Bennion K. A. (2013). Contributors to hypervigilance in a military and civilian sample. J. Interpers. Violence 28, 1672–1692. doi: 10.1177/0886260512468319, PMID: 23334188 PMC4157995

[ref27] Kuckertz J. M. Amir N. Boffa J. W. Warren C. K. Rindt S. E. Norman S. . (2014). The effectiveness of an attention bias modification program as an adjunctive treatment for post-traumatic stress disorder. Behav. Res. Ther. 63, 25–35. doi: 10.1016/j.brat.2014.09.002, PMID: 25277496 PMC4258474

[ref28] Lampit A. Hallock H. Valenzuela M. (2014). Computerized cognitive training in cognitively healthy older adults: a systematic review and meta-analysis of effect modifiers. PLoS Med. 11:e1001756. doi: 10.1371/journal.pmed.1001756, PMID: 25405755 PMC4236015

[ref29] Larsen S. E. Lotfi S. Bennett K. P. Larson C. L. Dean-Bernhoft C. Lee H. J. (2019). A pilot randomized trial of a dual n-back emotional working memory training program for veterans with elevated PTSD symptoms. Psychiatry Res. 275, 261–268. doi: 10.1016/j.psychres.2019.02.015, PMID: 30939398 PMC6508098

[ref31] Lewis C. Roberts N. P. Andrew M. Starling E. Bisson J. I. (2020). Psychological therapies for post-traumatic stress disorder in adults: systematic review and meta-analysis. Eur. J. Psychotraumatol. 11:1729633. doi: 10.1080/20008198.2020.1729633, PMID: 32284821 PMC7144187

[ref32] McGorry P. D. Hickie I. B. Yung A. R. Pantelis C. Jackson H. J. (2006). Clinical staging of psychiatric disorders: a heuristic framework for choosing earlier, safer and more effective interventions. Aust. N. Z. J. Psychiatry 40, 616–622. doi: 10.1080/j.1440-1614.2006.01860.x, PMID: 16866756

[ref33] Moher D. Hopewell S. Schulz K. F. Montori V. Gøtzsche P. C. Devereaux P. J. . (2010). Explanation and elaboration: updated guidelines for reporting parallel group randomised trials. BMJ 340:c869. doi: 10.1136/bmj.c869, PMID: 20332511 PMC2844943

[ref34] Motter J. N. Pimontel M. A. Rindskopf D. Devanand D. P. Doraiswamy P. M. Sneed J. R. (2016). Computerized cognitive training and functional recovery in major depressive disorder: a meta-analysis. J. Affect. Disord. 189, 184–191. doi: 10.1016/j.jad.2015.09.022, PMID: 26437233

[ref35] Nicholson A. A. Friston K. J. Zeidman P. Harricharan S. McKinnon M. C. Densmore M. . (2017). Dynamic causal modeling in PTSD and its dissociative subtype: bottom–up versus top–down processing within fear and emotion regulation circuitry. Hum. Brain Mapp. 38, 5551–5561. doi: 10.1002/hbm.23748, PMID: 28836726 PMC6866710

[ref36] Olff M. Polak A. R. Witteveen A. B. Denys D. (2014). Executive function in posttraumatic stress disorder (PTSD) and the influence of comorbid depression. Neurobiol. Learn. Mem. 112, 114–121. doi: 10.1016/j.nlm.2014.01.003, PMID: 24440596

[ref37] Organization WH (2022). International classification of diseases, 11th revision (ICD-11). Geneva: World Health Organization.

[ref38] Page M. J. McKenzie J. E. Bossuyt P. M. Boutron I. Hoffmann T. C. Mulrow C. D. . (2021). The PRISMA 2020 statement: an updated guideline for reporting systematic reviews. BMJ 372:n71. doi: 10.1136/bmj.n71, PMID: 33782057 PMC8005924

[ref39] Patel R. Spreng R. N. Shin L. M. Girard T. A. (2012). Neurocircuitry models of posttraumatic stress disorder and beyond: a meta-analysis of functional neuroimaging studies. Neurosci. Biobehav. Rev. 36, 2130–2142. doi: 10.1016/j.neubiorev.2012.06.003, PMID: 22766141

[ref40] Polak A. R. Witteveen A. B. Reitsma J. B. Olff M. (2012). The role of executive function in posttraumatic stress disorder: a systematic review. J. Affect. Disord. 141, 11–21. doi: 10.1016/j.jad.2012.01.001, PMID: 22310036

[ref41] Popescu M. Popescu E. A. DeGraba T. J. Hughes J. D. (2023). Cognitive flexibility in post-traumatic stress disorder: sustained interference associated with altered modulation of cortical oscillatory activity during task-switching. Neuroimage Clin. 37:103297. doi: 10.1016/j.nicl.2022.103297, PMID: 36563647 PMC9795531

[ref42] Post L. M. Zoellner L. A. Youngstrom E. Feeny N. C. (2011). Understanding the relationship between co-occurring PTSD and MDD: symptom severity and affect. J. Anxiety Disord. 25, 1123–1130. doi: 10.1016/j.janxdis.2011.08.003, PMID: 21899984 PMC3196268

[ref43] Rock P. L. Roiser J. P. Riedel W. J. Blackwell A. D. (2014). Cognitive impairment in depression: a systematic review and meta-analysis. Psychol. Med. 44, 2029–2040. doi: 10.1017/S0033291713002535, PMID: 24168753

[ref44] Rosen V. Ortiz N. F. Nemeroff C. B. (2020). Double trouble: treatment considerations for patients with comorbid PTSD and depression. Curr Treat Options Psych. 7, 258–274. doi: 10.1007/s40501-020-00213-z

[ref45] Rytwinski N. K. Scur M. D. Feeny N. C. Youngstrom E. A. (2013). The co-occurrence of major depressive disorder among individuals with posttraumatic stress disorder: a meta-analysis. J. Trauma. Stress. 26, 299–309. doi: 10.1002/jts.21814, PMID: 23696449

[ref46] Schäfer I. Gast U. Hofmann A. Knaevelsrud C. Lampe A. Liebermann P. . (2019). S3-Leitlinie Posttraumatische. Belastungsstörung: Springer.

[ref47] Schein J. Houle C. Urganus A. Cloutier M. Patterson-Lomba O. Wang Y. . (2021). Prevalence of post-traumatic stress disorder in the United States: a systematic literature review. Curr. Med. Res. Opin. 37, 2151–2161. doi: 10.1080/03007995.2021.1978417, PMID: 34498953

[ref48] Shipstead Z. Redick T. S. Engle R. W. (2012). Is working memory training effective? Psychol. Bull. 138, 628–654. doi: 10.1037/a0027473, PMID: 22409508

[ref49] Sterne J. A. Savović J. Page M. J. Elbers R. G. Blencowe N. S. Boutron I. . (2019). RoB 2: a revised tool for assessing risk of bias in randomised trials. BMJ 366:l4898. doi: 10.1136/bmj.l4898, PMID: 31462531

[ref50] Sterne J. A. Sutton A. J. Ioannidis J. P. Terrin N. Jones D. R. Lau J. . (2011). Recommendations for examining and interpreting funnel plot asymmetry in meta-analyses of randomised controlled trials. BMJ 343:d4002. doi: 10.1136/bmj.d4002, PMID: 21784880

[ref51] Williamson C. Baumann J. Murphy D. (2023). Exploring the health and well-being of a national sample of U.K. treatment-seeking veterans. Psychol. Trauma 15, 672–680. doi: 10.1037/tra0001356, PMID: 36222665

[ref52] Wolf E. J. Lunney C. A. Schnurr P. P. (2016). The influence of the dissociative subtype of posttraumatic stress disorder on treatment efficacy in female veterans and active duty service members. J. Consult. Clin. Psychol. 84, 95–100. doi: 10.1037/ccp0000036, PMID: 26167946 PMC4830387

